# Effects of COVID-19 on Sense of Smell: Human Factors/Ergonomics Considerations

**DOI:** 10.1177/0018720821990162

**Published:** 2021-02-01

**Authors:** E. Leslie Cameron, Per Møller, Keith S. Karn

**Affiliations:** 1 4224 Carthage College, Kenosha, Wisconsin, USA; 2 154917 Per Møller Consulting, Bagsværd, Denmark; 3 Human Factors in Context LLC, Philadelphia, Pennsylvania, USA

**Keywords:** olfaction, psychophysical methods, multi-modality displays, product design, universal design

## Abstract

**Objective:**

We review the effects of COVID-19 on the human sense of smell (olfaction) and discuss implications for human-system interactions. We emphasize how critical smell is and how the widespread loss of smell due to COVID-19 will impact human-system interaction.

**Background:**

COVID-19 reduces the sense of smell in people who contract the disease. Thus far, olfaction has received relatively little attention from human factors/ergonomics professionals. While smell is not a primary means of human-system communication, humans rely on smell in many important ways related to both quality of life and safety.

**Method:**

We briefly review and synthesize the rapidly expanding literature through September 2020 on the topic of smell loss caused by COVID-19. We interpret findings in terms of their relevance to human factors/ergonomics researchers and practitioners.

**Results:**

Since March 2020 dozens of articles have been published that report smell loss in COVID-19 patients. The prevalence and duration of COVID-19-related smell loss is still under investigation, but the available data suggest that it may leave many people with long-term deficits and distortions in sense of smell.

**Conclusion:**

We suggest that the human factors/ergonomics community could become more aware of the importance of the sense of smell and focus on accommodating the increasing number of people with reduced olfactory performance.

**Application:**

We present examples of how olfaction can augment human-system communication and how human factors/ergonomics professionals might accommodate people with olfactory dysfunction. While seemingly at odds, both of these goals can be achieved.

Attempts to answer the question of how human factors/ergonomics professionals can help with the ongoing COVID-19 pandemic have focused primarily on clinical environments (see Gurses et al., 2020). In this article we take a different approach, considering patients after recovery from the most severe symptoms of the disease who may suffer a reduction in, or loss of, sense of smell (olfaction). We demonstrate the importance of olfaction in human perception and performance, and encourage the human factors/ergonomics community to become more aware of the importance of human olfaction and consider taking action in two disparate ways:

- integrate olfaction into systems design as another means of human-system communication and
- accommodate the increasing number of people with reduced smell function.

While these two goals may seem contradictory, we feel both are achievable and important. Olfaction can be used as another form of redundant sensory coding, just as we use multiple forms of visual, auditory, and tactile coding in human-system communication.

## COVID-19 and Sense of Smell

At the time of submission of this review, dozens of studies had been published regarding smell loss in COVID-19 patients, with the earliest appearing in late March (Hopkins & Kumar, 2020) indicating that smell loss could be an early marker of the disease. Most of the data reported thus far come from self-report (e.g., Bagheri et al., 2020; Giacomelli et al., 2020; Lechien et al., 2020; Parma et al., 2020; Qiu et al., 2020; Speth et al., 2020; Yan et al., 2020), which is well known not to correlate with objective measures of smell function (Landis et al., 2003; Soter et al., 2008; Wehling et al., 2011). *Microsmia*—a subtle smell loss—often goes undetected, and even *anosmia*—complete smell loss—can go undetected. Thus, the extent of smell loss from COVID-19 may be underestimated (e.g., Tong et al., 2020). Indeed, one study that measured smell loss objectively found that 98% of COVID-19 patients had some smell loss although only 35% reported any smell dysfunction when asked (Moein, Hashemian, Mansourafshar, et al., 2020).

Although prevalence estimates have varied considerably, it is clear that many or even most COVID-19 patients have demonstrable smell loss. By mid-April 2020, the Center for Disease Control had officially listed “New loss of taste or smell” as a symptom of COVID-19 (https://www.cdc.gov/coronavirus/2019-ncov/symptoms-testing/symptoms.html).

The duration of smell loss in COVID-19 patients is still under investigation, but available data suggest that recovery typically occurs, if not completely, within days or weeks (Hopkins et al., 2020; Lee et al., 2020; Moein, Hashemian, Tabarsi, et al., 2020). Given the duration of the pandemic, no studies have yet been able to establish the long-term effects of COVID-19 on smell loss. Anecdotal evidence suggests that for some people, smell loss may be long-lasting (see, for example, posts at: https://www.facebook.com/groups/AbScentCovid19/). Even when recovery of function occurs, a later post-recovery parosmia—distortion of the perception of odors—has also been reported (Burges Watson et al., 2020).

Upper respiratory viral infections (“common colds”) are one of the primary triggers for permanent smell loss (Hawkes & Doty, 2009). Given that smell loss is a symptom of COVID-19 and that self-report may significantly underestimate the extent of the issue, the scale of the pandemic means that at any given time, many people will experience deficiencies in their sense of smell. Furthermore, even if persistent olfactory dysfunction affects only a small percentage of COVID-19 survivors, there may be a substantial number left with long-term smell deficits (Soler et al., 2020).

## Functions of Smell

Widespread loss of smell due to COVID-19 has increased awareness of how critical the chemical senses are to human behavior. Personal responses to such losses range from minor annoyance to major devastation; consider the end of a career for a chef.

Because humans rely so heavily on vision and audition, a majority of perceptual research over the past 100 years has focused on these senses. Similarly, both research and applied human factors/ergonomics work has focused on visual and auditory displays to engage these primary senses and facilitate human-system interaction. Human factors/ergonomics practitioners also exploit other sensory modalities such as vibro-tactile sense, kinesthesia, proprioception, and vestibular senses, but in less overt ways. For example, a driver may rely on kinesthesia and proprioception to learn the locations of controls in a car and rely on tactile sensation to discriminate between two differently shaped controls.

On the other hand, olfaction has generally received less attention from human factors/ergonomics professionals, aside from a mention in introductory textbooks of the addition of methyl mercaptan to natural gas, which is odorless and almost impossible to detect by itself. The important functions that olfaction plays in basic human behavior are often less obvious than those of vision and audition and therefore less appreciated. We rely on smell in important ways that relate to both quality of life and safety. Stevenson (2010) identified three classes of smell function—those related to ingestion (e.g., enjoyment of food, eating a well-balanced diet, avoiding unsafe foods), avoiding environmental hazards (e.g., gas leaks, fires), and social communication (e.g., recognizing kin). Furthermore, familiar, expected odors make us “feel at home” whereas novel, unexpected odors detected in well-known environments, make us uneasy (Köster et al., 2014). Although rarely acknowledged, olfactory problems are associated with a variety of negative outcomes (Erskine & Philpott, 2020; Miwa et al., 2001). Age-related deficits in smell function explain why many elderly persons complain that food lacks flavor and a disproportionate number die in accidental gas poisonings. These chemosensory deficits have been implicated in increased risk related to hazards such as food poisoning, fire, nutritional deficiencies, and hazardous chemical exposure (Santos et al., 2004 for a review, see Schiffman, 1997).

The sense of smell interacts with other sensory systems (see De Luca & Botelho, 2019 for a review). For example, the combination of smell and taste underlies flavor and our experience of food. Indeed, it is common for people to believe they have a taste loss when they are actually experiencing smell loss (Hawkes & Doty, 2009). Odors can be used to increase attentiveness and performance on a visual task requiring sustained attention (vigilance) without increasing stress (Dember et al., 1995; Warm et al., 1991). Though not a contributing factor to motion sickness, unpleasant smells, are perceived as even more unpleasant; and pleasant smells, more pleasant after motion-induced sickness (Paillard et al., 2014). Smell also plays an important role in emotional state (Herz, 2002; Hofer et al., 2020). The multi-billion-dollar fragrance and perfume industry speaks to the strength of the emotional effects of scents.

## Olfaction in System Design

Now that COVID-19 has awakened us to the fragility of our sense of smell, and given the important functions that olfaction serves, here we consider some possible ways in which human factors/ergonomics professionals could be thinking about olfaction. First, we address ways in which olfaction can be further harnessed as a means of human-system interaction and second, we address ways to accommodate people’s olfactory dysfunction. While these two goals may conflict at times, we feel that both are achievable.

### Smell: An Additional Channel of Human-System Communication

Since about 2010 there has been a small but steady increase in applied research on “multiple sensorial media” known as “mulsemedia” (see, for example, Ghinea et al., 2014; Kannan & Andres, 2010). Mulsemedia systems involve at least three senses (Ghinea et al., 2014)—increasingly including smell—as opposed to “multimedia” systems that typically integrate just audio and visual displays.

There is a long, but intermittent history of using smell as a means of communication between systems and human users, starting with the scent of roses delivered to audiences while watching a film of the Rose Bowl in 1906 (Logino, 1999 as cited in Kaye, 2001). The consideration of smell as an additional channel of human-system interaction is on the rise for both entertainment and productivity (e.g., Maggioni et al., 2018). For a review of the technology associated with olfactory interfaces and related human factors issues see Yanagida (2008).

Here we present some possible ways to integrate smell as an additional channel of communication in system design.

- Some odors are known to have a calming effect and can be used in stressful environments, such as emergency department waiting rooms. For example, pleasant, familiar odors reduce perceived stress and peripheral nervous system activity (Calvi et al., 2020; Glass & Heuberger, 2016; Joussain et al., 2014). These effects depend on learned associations (e.g., Christoffersen & Schachtman, 2016). Such effects might cause people to perceive dental treatment as more painful when they smell the typical eugenol (clove) odor in a dental clinic (Robin et al., 1999). Using other odors to mask this odor might reduce anxiety and pain of dental visits.
- Odors can influence consumer behavior, such as in *scent marketing*. Car dealerships exploit the “new car smell,” hotel chains employ distinctive scents, and many fashion shops use fragrances to influence customers’ behavior. Teerling et al. (1992) found that some odors, added to the ventilation systems of clothing stores in low concentrations, resulted in longer customer visits and had a positive effect on sales. Likewise, Holland et al. (2005) found that people exposed to a citrus-scented cleaning product demonstrated both increased mental access to the concept of cleaning and increased cleaning behavior. Although beyond the scope of this review, some research has suggested that odors can influence behavior even when presented at a level below conscious awareness (De Luca & Botelho, 2019; Li et al., 2007).
- Smells could be used to guide people with dementia around a memory care institution in which they live. Visual signage, which relies on reading and visual and semantic memory, might not work well for these people. Smells could be used by residents to help them find their own corridors by using characteristic smells for each corridor. This technique could also be used to guide blind people to a corridor or room.
- Smells can help to “set the stage” for various tasks for groups of mentally disabled people. For example, food odors from the kitchen can be used to help prepare people for mealtime by setting up expectations and generating the appetite and cephalic responses that prepare the digestive system. Chlorine odor has been used in a Dutch residential facility to set the stage for changing into a swimsuit before a trip to the swimming pool (E.P. Köster, personal communication, June 25, 2020).

All of the above effects hold great potential for human factors applications.

### Accommodating People With Smell Dysfunction

Human factors/ergonomics professionals strive to consider all potential users of systems that we help design. We are trained to accommodate people with a wide range of anthropometric dimensions and physical, visual, auditory, and cognitive abilities. Given the smell loss that many people will experience as a result of COVID-19, and the risk of harm that comes with this loss, we are now called upon to consider how the human factors/ergonomics community might help. We can increase awareness of deficits in smell function and the implications it has for safety and quality of life. Many people are unaware of their olfactory deficits. So, in many cases, just being made aware or reminded of olfactory deficits may allow people to cope more effectively with their sensory losses. Here are some avenues for exploration in which we may be able to help people cope more directly with olfactory deficits:

- Increase reminders both within the devices themselves and in public service announcements to check batteries in smoke detectors to make up for decreases in sensitivity to smelling smoke.
- Increase size and contrast of expiration dates or provide other salient, visual indications when food is spoiled.
- Increase prominence of nutrition labels (see Goldberg et al., 1999) and awareness of nutrient intake to compensate for nutritional inadequacies associated with the loss of smell.
- Recruit people with olfactory dysfunction when conducting usability studies on systems where smell is used as a means of human-system communication.
- Use objective measures of olfactory function when employees are recovering from smell loss to determine when they are able to return to work where sense of smell is critical for task performance (e.g., gas line maintenance, food production/quality control).
- Provide other indicators (e.g., visual) as reminders to launder soiled clothes and change diapers.
- Provide visual or auditory indicators that a mechanical device is overheating or malfunctioning in other ways that might normally be detected by smell.
- Provide visual indicators that chemicals such as chlorine are reaching unhealthy levels (either too high or too low) in a swimming pool or water treatment system.
- Improve surgical devices by providing visual and auditory feedback to surgeons who no longer can smell burning tissue or other relevant smells.
- Provide more salient visual indicators on gas stoves to indicate when they are on and releasing gas, make gas stove valves less susceptible to accidental activation (Pollack, 2019), and encourage people to use electronic gas detectors (Wogalter & Laughery, 2011).
- Develop a gauge to measure spiciness of food.

This is not intended as an exhaustive list. Rather we encourage readers to consider these and other ways that human factors/ergonomics professionals can help guide user interface design to accommodate COVID-19 patients who have lost their sense of smell.

## Measuring Sense of Smell

There are many reasons that human factors/ergonomics professionals may need to assess a person’s sense of smell. Here are some examples:

- Screening participants for usability testing of a medication delivery system in which smell indicates a leak.
- Determining if a mine worker is fit for work in an underground environment in which an odor is added to ventilation air to signal emergency evacuation.
- Testing to determine the appropriate strength of an odor intended to elicit a specific response in a new system design.

There are a number of commercially available, objective tests to assess sense of smell, but by far the most commonly used tests are the University of Pennsylvania Smell Identification Test, UPSIT (Doty et al., 1984) from Sensonics (www.sensonics.com) and the Screening 12 Test kit from Burghart (https://smelltest.eu/en/burghart-sniffin-sticks-burghart-smelltests/). We encourage use of objective measures of smell function whenever possible, given that self-report is unreliable.

## Conclusion

In conclusion, the COVID-19 pandemic is causing significant loss of smell in many people. The scale of the pandemic means that at any given time, many people will experience deficiencies in their sense of smell, and some may experience permanent dysfunction. We encourage the human factors/ergonomics professional community to (1) be aware of the importance of the sense of smell for human behavior and of the COVID-19-related deficits in smell function, (2) consider how smell deficits impact users of products and systems that we help develop, (3) consider ways to accommodate people with such sensory disabilities, and (4) consider new ways of using smell as an additional channel of human-system communication.

## Key Points

- COVID-19 is known to reduce the olfactory sense (smell) in people who contract the disease. The duration of the loss is not well characterized at this time, but some may experience permanent dysfunction in the sense of smell.
- Human factors/ergonomics professionals should be aware of the increased number of people with olfactory dysfunction and consider ways to accommodate them.
- While the sense of smell is not commonly thought of as a primary means of human-system communication, we rely on smell in many important ways that relate to both quality of life and safety.
- Olfaction has received relatively little attention from human factors/ergonomics professionals. Product research and development teams have just begun to tap into the sense of smell to augment human system communication.
